# Mitochondrial Calcium Regulation of Redox Signaling in Cancer

**DOI:** 10.3390/cells9020432

**Published:** 2020-02-12

**Authors:** Céline Delierneux, Sana Kouba, Santhanam Shanmughapriya, Marie Potier-Cartereau, Mohamed Trebak, Nadine Hempel

**Affiliations:** 1 Department of Cellular and Molecular Physiology, The Pennsylvania State University College of Medicine, 500 University Dr., Hershey, PA 17033, USA; celine.delierneux@gmail.com (C.D.); mtrebak@pennstatehealth.psu.edu (M.T.); 2 Inserm U1069 Nutrition, Croissance et Cancer, Université de Tours, 10 Boulevard Tonnellé, 37032 Tours, France; sana.kouba@hotmail.com (S.K.); marie.potier-cartereau@univ-tours.fr (M.P.-C.); 3 Department of Medicine, The Pennsylvania State University College of Medicine, 500 University Dr., Hershey, PA 17033, USA; ssanthanam@pennstatehealth.psu.edu; 4 Department of Pharmacology, and Obstetrics and Gynecology, The Pennsylvania State University College of Medicine, 500 University Dr., Hershey, PA 17033, USA

**Keywords:** mitochondrial Ca^2+^ transport, ROS, redox signaling, mitochondrial ROS, cancer

## Abstract

Calcium (Ca^2+^) uptake into the mitochondria shapes cellular Ca^2+^ signals and acts as a key effector for ATP generation. In addition, mitochondria-derived reactive oxygen species (mROS), produced as a consequence of ATP synthesis at the electron transport chain (ETC), modulate cellular signaling pathways that contribute to many cellular processes. Cancer cells modulate mitochondrial Ca^2+^ ([Ca^2+^]m) homeostasis by altering the expression and function of mitochondrial Ca^2+^ channels and transporters required for the uptake and extrusion of mitochondrial Ca^2+^. Regulated elevations in [Ca^2+^]m are required for the activity of several mitochondrial enzymes, and this in turn regulates metabolic flux, mitochondrial ETC function and mROS generation. Alterations in both [Ca^2+^]m and mROS are hallmarks of many tumors, and elevated mROS is a known driver of pro-tumorigenic redox signaling, resulting in the activation of pathways implicated in cellular proliferation, metabolic alterations and stress-adaptations. In this review, we highlight recent studies that demonstrate the interplay between [Ca^2+^]m and mROS signaling in cancer.

## 1. Introduction

Calcium (Ca^2+^) is a major second messenger in cellular signaling. Ca^2+^ signaling regulates many physiological functions, including muscle contraction, neuronal excitability, cell migration, and growth. In order to maintain a delicate regulation of Ca^2+^ homeostasis, various plasma membrane and organellar Ca^2+^ channels, exchangers and transporters are needed [1]. The majority of intracellular Ca^2+^ is stored in the endoplasmic or sarcoplasmic reticulum (ER/SR). However, it is also known that dynamic organelles such as the mitochondria can play a major role in buffering and shaping cytosolic Ca^2+^ [2]. Depending on the distribution of mitochondria inside the cell, their ability to act as Ca^2+^ buffers has different consequences. For example, mitochondria can be located in regions close to the plasma membrane (PM), around the nucleus or at the periphery of the cell [3], where they can be independently activated by specific spatiotemporal patterns of cytosolic Ca^2+^ signals and can therefore participate in the local regulation of Ca^2+^ homeostasis [4]. Moreover, several cellular functions rely on the transport of Ca^2+^ cations into the mitochondrial matrix, such as the tricarboxylic acid cycle (TCA), adenosine triphosphate (ATP) production, and reactive oxygen species (ROS) generation [5]. A consequence of facilitating mitochondrial metabolism and oxidative phosphorylation (OXPHOS) is that [Ca^2+^]m can in turn participate in mitochondrial ROS (mROS) generation, which takes place at the electron transport chain (ETC) [6]. This implicates [Ca^2+^]m flux and mitochondrial Ca^2+^ transport as important regulators of mROS generation and redox signaling. In addition to physiologically necessary fluctuations in [Ca^2+^]m, excess [Ca^2+^]m can also determine cell fate, since mitochondrial Ca^2+^ overload induces the opening of the mitochondrial permeability transition pore (mPTP), which leads to mitochondrial membrane depolarization, elevated mROS, and the release of cytochrome *c*, to activate pro-apoptotic factors [7]. Consequently, a precise regulation between [Ca^2+^]m uptake and extrusion is necessary to ensure proper cellular functions. 

In this review, we briefly describe the composition and regulation of [Ca^2+^]m transport; we discuss some of the recent discoveries regarding the role of [Ca^2+^]m in cancer cells; and focus on how [Ca^2+^]m transport is an important regulatory mechanism by which cancer cells elicit mROS-dependent pro-tumorigenic signaling.

## 2. Mitochondrial Calcium (Ca^2+^) Transport

### 2.1. Mitochondrial Ca^2+^ Uptake 

Calcium (Ca^2+^) uptake into the mitochondrial matrix from the cytoplasm occurs at the mitochondria-associated membranes (MAMs), where the ER is juxtaposed within 10 to 25 nm to mitochondria in microdomains containing tethering protein and channels, which help Ca^2+^ exchange between these two organelles (reviewed in [8,9]). In order to reach the mitochondrial matrix, cytosolic Ca^2+^ has to cross two membranes: the outer mitochondrial membrane (OMM) and the inner mitochondrial membrane (IMM). In the OMM, the voltage-dependent anion channel proteins (VDACs) are highly permeable to Ca^2+^ and mediate Ca^2+^ transport into the intermembrane space (IMS). VDACs are also involved in cell metabolism by transporting ATP and other small metabolites across the OMM [10]. Three isoforms of the VDACs proteins have been identified (VDAC1, VDAC2, and VDAC3) of which, VDAC1 is the most abundantly characterized and is considered as the main Ca^2+^ transport channel [11,12]. The IMM is folded into cristae and is much less permeable to ions and small molecules than the outer membrane. In response to the electrochemical gradient (ΔΨm) across the IMM, Ca^2+^ ions therefore cross the IMM through a Ca^2+^ channel, termed the mitochondrial Ca^2+^ Uniporter (MCU). Molecular and genomic characterization revealed that MCU functions as a multimeric complex. 

MCU is the pore-forming subunit and its knockdown abolishes mitochondrial Ca^2+^ uptake, while its overexpression strongly enhances mitochondrial Ca^2+^ uptake [13]. The MCU protein, previously known as CCDC109A, is a 40 KDa protein characterized by two transmembrane domains with N-and C-termini facing the matrix [14]. The other component of the channel-forming subunits is MCUb, previously known as CCDC109B. MCUb is a protein of approximately 33 KDa and its topology is similar to MCU, with two transmembrane domains and both *N*- and *C*-termini are extended into the matrix. MCUb is a paralog of MCU but has no channel activity. Studies showed that its overexpression reduced [Ca^2+^]m, suggesting that it might be a negative regulator of MCU [15]. The third channel-forming subunit is the essential MCU regulator (EMRE), also known as SMDT1. EMRE is a single-pass transmembrane protein of 10 KDa. Its *N*-terminus is located in the mitochondrial matrix, where it can interact with MCU, while its *C*-terminus is in the IMS, where it can associate with the MCU gatekeeping subunit protein—mitochondrial Ca^2+^ uptake protein 1 (MICU1) [16]. MICU1 functions in heterodimeric associations with its homologue, MICU2. EMRE is required for MCU interaction with MICU1 and MICU2, and loss of EMRE induces a reduction of mitochondrial Ca^2+^ uptake [17]. 

Ca^2+^-binding domains have not been identified in the MCU protein sequence, suggesting that MCU is unable to regulate its own activity and requires MICU proteins. MICU1 is a single transmembrane membrane protein of approximately 54 KDa and binds to its paralog MICU2 to form a 95 KDa active heterodimer [18]. MICU1/2 associates with MCU and is characterized by the presence of Ca^2+^-binding EF-hand domains [19]. Loss-of-function studies have demonstrated that MICU1 and MICU2 act as gatekeepers of the uniporter, therefore setting the threshold concentration of Ca^2+^ for MCU activation [19,20]. MICU1 and MICU2 keep MCU closed at resting cytosolic Ca^2+^ concentrations but allow MCU to open when cytosolic Ca^2+^ reaches sub-µM levels. Ca^2+^ uptake into mitochondria is thereby triggered only at high concentrations, preventing its accumulation inside the matrix under unstimulated conditions [20,21,22]. At nM cytosolic Ca^2+^ concentrations, it was found that MICU1 alone is sufficient to inhibit MCU activity, while at higher cytosolic Ca^2+^ concentrations (μM range) MICU1 enhances MCU activity, and requires the presence of MICU2 [18,23]. Another study demonstrated that MICU1 knockdown is followed by the loss of MICU2 protein. This loss of both proteins caused Ca^2+^ overload in the mitochondrial matrix and inhibition of MCU maximal activation due to loss of cooperativity. Other regulatory elements of the complex have been described, including MICU3 [24,25] and MICU1.1, a splice variant of MICU1 with a higher binding affinity to Ca^2+^ [26]. MICU3 and MICU1.1 are tissue-specific and are expressed in the central nervous system and skeletal muscle, respectively. However, their role in regulating MCU activity is less well established.

Another MCU regulator is the mitochondrial regulator MCUR1, which is approximately 40 KDa and consists of two transmembrane domains, with the N-and C-terminal spanning to the IMS and a large portion of the protein facing the matrix. MCUR1 knockdown abrogates Ca^2+^ uptake and its overexpression enhances [Ca^2+^]m entry [27]. It is not well known how MCUR1 regulates MCU and the hypothesis of a direct interaction is still under debate; but one study suggested that MCUR1 binds to both MCU and EMRE, and serves as a scaffold protein of the complex, since loss of MCUR1 perturbs the complex’ assembly [28].

### 2.2. Mitochondrial Ca^2+^ Extrusion

In order to maintain proper [Ca^2+^]m homeostasis, Ca^2+^ cations must be extruded from the mitochondrial matrix. The Mitochondrial Na^+^/ Ca^2+^/Li^+^ exchanger (NCLX), also known as solute carrier family 8-member B1 (SLC8B1) is one of the main proteins responsible for Ca^2+^ efflux from the matrix [29,30]. NCLX activity consists of extruding one Ca^2+^ from the mitochondria and importing three Na^+^. It is expressed ubiquitously among tissues and its molecular weight is approximately 60 kDa. NCLX has two domains: α1 and α2. containing six transmembrane segments each. When NCLX was first cloned in 2004, it was proposed to be localized in the endoplasmic reticulum (ER) or at the PM [31], but later found to be enriched in mitochondria and localized in the IMM [32]. Palty et al., showed that NCLX overexpression increased the Ca^2+^ efflux to the IMS, while its downregulation led to a decrease of [Ca^2+^]m efflux. This effect was also confirmed by pharmacological inhibition of NCLX, using the CGP-37157 compound. Unlike other members of the NCX family, NCLX does not contain an allosteric Ca^2+^-binding site [33,34]. Rather than direct Ca^2+^ sensing, several studies demonstrated that the PTEN-induced putative kinase 1 (PINK1) is one of the main regulators of [Ca^2+^]m efflux [35], mainly through the protein kinase A (PKA) phosphorylation site at serine 258 (Ser258) which is located in the long loop connecting the α1 and α2 domains of NCLX [36]. Another exchanger is critical for proper NCLX activity, which is the Na^+^/H^+^ exchanger NHE. NHE is responsible for Na^+^ efflux into the cytosol, therefore maintaining Na^+^ gradients across the mitochondrial membrane [37] and Ca^2+^ homeostasis in mitochondria [38]. Another protein also implicated in Ca^2+^ extrusion from mitochondria is Letm1, also known as the mitochondrial Ca^2+^/H^+^ antiporter. This protein mediates the proton-dependent Ca^2+^ efflux from mitochondria [39]. Unlike NCLX, Letm1 is not a major factor for [Ca^2+^]m extrusion, but the activity of Letm1 is crucial for maintaining the tubular shape and cristae organization [40]. We refer the reader to the following review articles for a more comprehensive coverage of the mechanisms and proteins involved in mitochondrial Ca^2+^ transport [41,42].

## 3. Mitochondrial Ca^2+^, Reactive Oxygen Species (mROS) and Cancer

### 3.1. Mitochondrial Reactive Oxygen Species (mROS)

**Figure 1:**
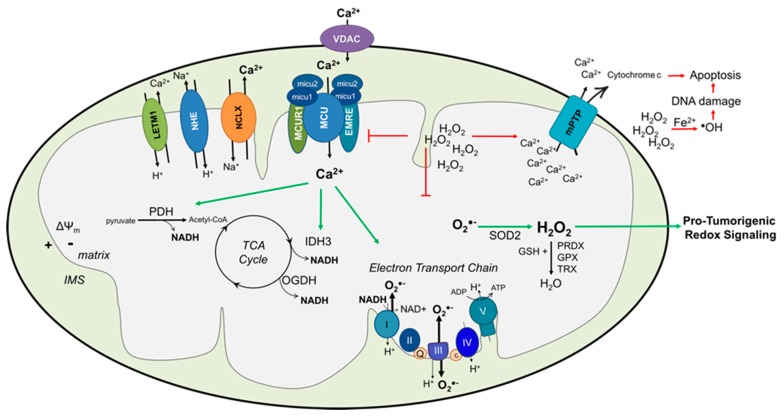
Cancer cells alter mitochondrial Ca^2+^ dynamics to enhance pro-tumorigenic mROS signaling (green arrows). Altered expression or post-translational regulation of proteins involved in mitochondrial Ca^2+^ transport, including MCU complex components MCU, MCUR1, and the gatekeeper MICU1, results in enhanced [Ca2+]m across the inner mitochondrial membrane. Ca2+ within the mitochondrial matrix activates dehydrogenases PDH, IDH3 and OGDH, resulting in enhanced NADH production and subsequent increase in oxidative phosphorylation at the electron transport chain. Increased flux through the electron transport chain increases superoxide production, which is rapidly converted to H_2_O_2_, which is responsible for redox signaling. Tumor cells regulate mitochondrial Ca^2+^ transport and antioxidant enzymes to prevent deleterious [Ca^2+^]m and mROS accumulation (red arrows; IMS: intermembrane space, ΔΨm: mitochondrial membrane potential, TCA: tricarboxylic acid, O_2_^•−^: superoxide, H_2_O_2_: hydrogen peroxide, ^•^OH: hydroxyl radical, SOD: superoxide dismutase, NADH: nicotinamide adenine dinucleotide, MCU: mitochondrial Ca^2+^ uniporter, VDAC: voltage-dependent anion channel, mPTP: permeability transition pore, Letm1: H^+^/Ca^2+^ exchanger, GSH: glutathione, PRDX: peroxiredoxin, GPX: glutathione peroxidase, TRX: thioredoxin reductase).

Cellular ROS include free radicals such as superoxide (O_2_^•−^) and hydroxyl radical (^•^OH), and non-radical species such as hydrogen peroxide (H_2_O_2_). These are generated at both the membrane, through NADPH-dependent oxidases (NOX/DUOX), and in the mitochondria, by the incomplete one-electron reduction of oxygen at respiratory chain complexes I and III [43,44] (Figure 1). ROS are generated in both physiological and pathological conditions. In physiological conditions, the concentration of ROS is highly regulated at both the level of generation and scavenging. Surges of mitochondrial ROS (mROS) are involved in cell death pathways, including apoptosis and necrosis. Moreover, subsequent generation of the highly reactive ^•^OH is responsible for macromolecular oxidative damage, including DNA and cellular damage. However, at sub-lethal levels, mROS also have important roles in signaling. Physiological changes in mROS initiate a wide variety of cellular responses, such as regulation of phosphatase/kinase signaling pathways, mitochondrial fission, autophagy, hypoxia, antioxidant defense adaptations, and regulation of aging-related mechanisms [45]. When the balance between ROS production and clearance is broken, mROS have been demonstrated to contribute to various pathologies such as neurodegenerative disorders [46], diabetes [47], cardiovascular diseases [48], and cancer [49]. This includes oxidative stress (large surges of ROS) which eventually lead to cell death, but may also involve sub-lethal changes that result in redox signaling events advantageous to disease progression. Tumor cells can operate under a higher ROS steady state compared to their normal or less metastatic counterparts [50,51,52,53]. This has been shown to be the result of changes in both the underlying genomic signature of tumor cells, such as Ras mutations, and alterations in the mitochondrial DNA, where accumulation of mtDNA mutations can increase steady state mROS levels in tumor cells (reviewed in [49]). Moreover, changes such as hypoxia, mitochondrial shape changes, alterations in the antioxidant enzyme milieu and cellular signaling pathways can all contribute to increased mROS in tumor cells [49,51]. In Section 3.3., we highlight several examples of mROS-mediated redox signaling that are important for tumorigenesis and cancer progression, and are specifically regulated by an altered mitochondrial Ca^2+^ milieu ([Ca^2+^]m) in cancer cells.

### 3.2. Mitochondrial Ca^2+^ Regulates Mitochondrial Metabolism and Reactive Oxygen Species (mROS) Generation

The role of regulated mitochondrial Ca^2+^ uptake and extrusion in cellular signaling is likely two-fold. First, the buffering capacity of mitochondria has been implicated in regulating cytosolic Ca^2+^ levels to influence Ca^2+^-dependent signaling outside of the mitochondria, such as the regulation of NFAT (nuclear factor of activated T-cells signaling) [54,55]. Second, regulated changes in [Ca^2+^]m, as eluded to above, influence the function of mitochondrial enzymes. This can alter cellular signaling due to effects on metabolism and metabolite pools, and due to the generation of mROS. Here we focus on the role of mitochondrial Ca^2+^ in regulating mROS in the context of cancer.

One of the major signaling mechanisms that control [Ca^2+^]m dynamics is store-operated Ca^2+^ entry (SOCE). SOCE is physiologically initiated upon agonist binding to Gq-protein coupled receptors at the plasma membrane. This leads to the activation of phospholipase C and breakdown of phosphatidylinositol 4,5-bisphosphate (PIP2) into inositol 1,4,5-trisphosphate (IP3) and diacylglycerol (DAG). IP3 binds to its receptor (IP3R) in the ER, leading to the release of Ca^2+^ from ER stores [56,57]. Ca^2+^ depletion is sensed by the ER-based stromal interaction molecules (STIM1 or STIM2), which initiate Ca^2+^ influx from the extracellular environment through Ca^2+^ release activated Ca^2+^ channels (CRAC) encoded by Orai proteins [58]. During SOCE, mitochondria can take up Ca^2+^ released from the ER, or entering through the plasma membrane, or both. 

Ca^2+^ influx into the mitochondrial matrix activates at least three key enzymes of cellular metabolism, including pyruvate dehydrogenase (PDH), NAD^+^ isocitrate dehydrogenase (IDH3), and 2-oxoglutarate dehydrogenase (OGDH) [59,60,61] (Figure 1). 

Pyruvate dehydrogenase (PDH) is located in the mitochondrial matrix and catalyzes a key reaction upstream of the TCA cycle. The last product of glycolysis, pyruvate, is transported in the mitochondrial matrix, where it is converted into Acetyl-CoA, which supplies the TCA cycle. This reaction is catalyzed by the E1 subunit of the PDH complex dehydrogenase complex, activity of which is regulated by phosphorylation. E1 phosphorylation by pyruvate dehydrogenase kinase (PDK) inactivates the PDH enzyme, while its de-phosphorylation activates it. Ca^2+^ regulates the activity of pyruvate dehydrogenase phosphatase (PDP) by binding it within a complex with the PDH L2 domain. High [Ca^2+^]m stimulates the activity of PDP, favoring PDH de-phosphorylation and thus its activity [59]. Accordingly, the reduction of mitochondrial Ca^2+^ uptake can increase the phosphorylated form of PDH, leading to its inactivation. This leads to us to an apparent paradox regarding the observed increases in [Ca^2+^]m observed in tumor cells. Given that many cancer cells exhibit increased PDK activity, which inhibits PDH and drives metabolism towards aerobic glycolysis [62], it remains to be determined if and how increased [Ca^2+^]m decreases PDH phosphorylation in the context of underlying genetic changes that increase PDK activity in cancer cells.

Isocitrate dehydrogenase (IDH3) catalyzes the oxidative decarboxylation of isocitrate to produce α-ketoglutarate in the TCA cycle. The product of this reaction, NADH, is an essential reducing equivalent for the ETC. Rises in ATP/ADP and NADH/NAD^+^ ratios inhibit the activity of IDH3, while high [Ca^2+^]m stimulate the enzyme. IDH3’s Kd for Ca^2+^ varies from 5 to 50 μM depending on the ATP/ADP ratio [59,63] and the enzyme becomes more sensitive to Ca^2+^ when the NADH/NAD^+^ ratio decreases [60]. A lack in IDH3 activity is usually lethal to cells, due to a loss of the TCA cycle. Conversely, increased IDH3 expression and activity has been observed in tumors and has been shown to drive TCA turn-over, mitochondrial ETC function and mROS production [64,65]. Although a direct link between elevated [Ca^2+^]m and enhanced IDH3 activity has not been made in cancer cells, it is possible that increased IDH3 expression and enhanced IDH3 activity via [Ca^2+^]m might cooperate to drive mROS production in tumors. Interestingly, tumor cells are able to run the TCA cycle in reverse via IDH2, and to utilize reductive carboxylation to generate citrate and subsequently NADPH and acetyl CoA, important substrates for glutathione reduction and fatty acid synthesis, respectively [66]. Whether or not enhanced [Ca^2+^]m-driven IDH3 activity opposes or is inhibited by enhanced reductive carboxylation in cancer cells requires further investigation. 

Oxoglutarate dehydrogenase (OGDH), catalyzes the next reaction of the TCA cycle, converting α-ketoglutarate to succinyl-CoA, and generating NADH. Similar to IDH3, its activity can regulate ETC function by providing the electron donor NADH. OGDH activity is enhanced by elevated [Ca^2+^]m [59,63,67,68], while its activity is inhibited by increases in succinyl-CoA/CoA and NADH/NAD^+^ ratios [69]. When these ratios are low, the enzyme sensitivity to Ca^2+^ rises. OGDH activity may therefore contribute to increased TCA cycle flux, ETC function and mROS generation in tumor cells. The lethality of inhibiting mitochondrial Ca^2+^ import in cancer cells can be rescued by dimethyl-α-ketoglutarate supplementation [70], demonstrating the interplay between the TCA cycle and [Ca^2+^]m. Since OGDH is an important regulator of α-ketoglutarate levels, which is a cofactor for demethylases, OGDH activity can therefore affect epigenetic transcriptional regulation. Whether OGDH activation by [Ca^2+^]m alters the epigenome in tumor cells has not been directly investigated. 

Given that [Ca^2+^]m activates the above metabolic enzymes, it is generally concluded that [Ca^2+^]m directly mediates the synthesis of the reduced substrate NADH of the respiratory electron transport chain (ETC), and increases OXPHOS (Figure 1) [71,72]. Although the mechanisms are not as well understood, [Ca^2+^]m has also been shown to directly affect proton pumping and ATP synthase activity of the ETC (for review see [73]). A consequence of increased mitochondrial metabolism and OXPHOS is mitochondrial O_2_^•−^ generation, which takes place at complexes I and III of the ETC [6,74] (Figure 1). [Ca^2+^]m has therefore been implicated as a key player in mROS production, which is correlated with metabolic rate [75]. While increased [Ca^2+^]m is clearly associated with mROS production in cancer cells, it should be noted that a direct link to alterations in metabolic enzyme activity has not been directly investigated in most tumor cell studies. 

In addition to the above controlled increases in mROS as a consequence of [Ca^2+^]m-regulated metabolism, excess Ca^2+^ influx into the mitochondria can also be detrimental to mitochondrial function. Too much [Ca^2+^]m results in a decrease in the mitochondrial membrane potential (ΔΨm), and can activate the mitochondrial permeability transition pore (mPTP) to induce Ca^2+^ and cytochrome *c* efflux, mitochondrial swelling, and break-down of the ΔΨm, which are important aspects of apoptosis initiation (Figure 1). In order to avoid lethal surges in mROS and mitochondrial oxidative stress resulting from an imbalance between ROS generation and detoxification, cells can set several mechanisms to neutralize mitochondria-generated ROS. O_2_^•−^ can dismute to hydrogen peroxide (H_2_O_2_) spontaneously or through enzymatic dismutation by the matrix manganese superoxide dismutase (MnSOD or Sod2) or copper/zinc superoxide dismutase (CuZnSOD or Sod1) in the IMS or the cytosol [76,77]. While mitochondria generated O_2_^•−^ is not membrane permeable, H_2_O_2_, on the other hand, is more stable, has much longer half-life than O_2_^•−^, and can pass more readily through membranes [78,79]. Therefore, H_2_O_2_ is considered as the “redox second messenger”. H_2_O_2_ levels are regulated by catalase, glutathione peroxidase, and thioredoxin peroxidase systems catalyzing the reduction of H_2_O_2_ to water (H_2_O) and oxygen (O_2_) [80,81]. H_2_O_2_ can also react with metal ions, such as Fe^2+^, via the Fenton reaction, to generate highly reactive hydroxyl radicals responsible for DNA oxidation, RNA or protein damage [82,83]. Taken all together, [Ca^2+^]m homeostasis, as well as well-adjusted ROS production and scavenging, are necessary to maintain a redox homeostasis that is required for normal function of the cell. In pathophysiological settings, including tumor cells, an imbalance of [Ca^2+^]m and mROS due to altered [Ca^2+^]m can therefore have different consequences on cell function. 

### 3.3. Reactive Oxygen Species (mROS) and Mitochondrial Redox Signaling in Cancer

Investigators have long noticed that cancer cells have higher production of ROS compared to normal cells [53]. While large surges of ROS contribute to tumorigenesis through DNA damage, sub-lethal levels of ROS are capable of activating signaling pathways that regulate cancer cell growth and progression [53,84,85]. One of the mechanisms by which ROS can regulate cell signaling is via cysteine oxidation. H_2_O_2_, for instance, inhibits the activity of the tumor suppressor PTEN (phosphatase and tensin homolog) by oxidizing its active site cysteine residues, causing the formation of a disulfide bond. This prevents PTEN from inactivating the PI3K (phosphoinositide 3-kinase) signaling pathway [49]. The PI3K-Akt cascade is an essential growth factor response pathway, which is upregulated in many cancers to induce proliferation and survival [86]. Another example is the oxidation of cysteines in the proto-oncogene Src [87,88]. Oxidation commonly activates kinases, and Src oxidation promotes cancer cell migration and metastasis—phenotypes that could be prevented when mROS were scavenged [89]. 

Elevated mROS also contribute to oxidative stress-resistance. Tumor cells are thus able to adapt to elevated ROS and prevent buildup of lethal surges in ROS by upregulating protective antioxidant pathways. A well-studied example of this in cancer cells is the transcriptional regulation of antioxidant enzymes by Nrf2 (nuclear factor erythroid-derived 2-like 2; NFE2L2) [90]. Nrf2 stabilization depends on ROS-mediated oxidation of cysteine residues on KEAP1 (Kelch-like ECH-associated protein 1), which is a negative regulator of Nrf2 [91]. The ability for tumor cells to deal with excess ROS is especially important during metastatic progression. For example, inhibiting NADPH production, an important cofactor required for glutathione reduction, inhibited melanoma metastasis but had no effect on primary tumor growth [92]. It was found that metastatic cells have higher levels of NADPH than primary tumor cells, and are therefore better equipped to handle increases in ROS during metastasis. Similarly, in a mouse model of malignant melanoma, antioxidant treatment, including the use of *N*-acetyl-cysteine, a glutathione precursor, promoted metastasis and cell invasion [93]. Taken together, these data highlight that cancer cells are capable of maintaining ROS levels within a range that is necessary for their proliferation and progression without causing cytotoxicity. 

Mitochondrial ROS are also elevated in response to hypoxia. As tumors grow, insufficient blood supply results in tumor regions where the oxygen concentration is significantly lower than in healthy tissues. Therefore, cancer cells activate the hypoxia stress response pathway, allowing them to metabolically adapt to the reduced oxygen microenvironment. The pathway consists of three hypoxia-sensitive α subunits (HIF1α, HIF2α, and HIF3α) that heterodimerize with HIF1β subunit and activate transcription [94]. It was shown that mROS are required to stabilize HIFα subunits under hypoxia using cells that lack the expression of mitochondrial DNA and ability to produce mROS [95]. These results were later confirmed by pharmacological inhibition of the ETC by rotenone or antimycin A, pointing to an important interplay between mROS production and HIFα stabilization under hypoxia [49,74]. The mROS-HIFα axis is an important regulator of metabolic adaptations. Activation of HIF1α promotes the expression of enzymes and transporters to increase glycolysis and glycolytic flux [96,97,98]. HIF1α in turn can also lead to increased mROS production by regulating microRNA-210 that targets ISCU1/2 (iron-sulfur cluster scaffold homolog) and COX10 (cytochrome *c* oxidase assembly protein), two important elements of the ETC [99,100]. In addition, mROS can change metabolism through Nrf2 activation, which is responsible for redirecting glucose and glutamine into anabolic pathways under the constitutive activation of the PI3K-Akt signaling cascade [101]. mROS can also oxidize the Cys-358 residue of the glycolytic enzyme pyruvate kinase M2 (PKM2) as it has been shown in lung cancer cells, where the oxidation of PKM2 under hypoxia induced an increased pentose phosphate pathway flux, glutathione levels and proliferation [102,103]. Another example is the activation of AMPK in response to the overexpression of manganese superoxide dismutase (MnSOD/SOD2) and consequential increases in mitochondrial H_2_O_2_ production in breast cancer cells [104]. Although we only provide a few examples from an expanding field, it is now well established that ROS from the mitochondria promote pro-tumorigenic redox-signaling, stress adaptations, and metabolic reprograming of tumor cells. 

## 4. Interplay between Mitochondrial Ca^2+^ and Reactive Oxygen Species (mROS) in Cancer 

Multiple investigations have highlighted a key role for alterations in cellular Ca^2+^ homeostasis in tumor cell proliferation, apoptosis resistance, tumor development, and metastasis [105]. Mechanisms that link [Ca^2+^]m homeostasis with tumor growth and progression are starting to emerge, in particular after the molecular identification of the proteins responsible for mediating mitochondrial Ca^2+^ uptake and extrusion, and the findings that their expression is often altered in tumor specimens. In this section, we highlight recent findings demonstrating that mitochondrial Ca^2+^ transport has important roles in tumorigenesis and cancer progression by altering mROS. We should note that few studies directly compare [Ca^2+^]m and mROS levels between cancer and matching normal tissues and cells. Although, it is generally assumed that cancer cells have altered [Ca^2+^]m levels compared to normal cells based on changes in expression of [Ca^2+^]m transport mechanisms. As seen in Table 1, many studies rely on genetic manipulation to establish a pathological role for the alterations of mitochondrial [Ca^2+^]m transport mechanisms and concomitant mROS changes, or demonstrate that these changes are exacerbated in cells with a more malignant phenotype. 

### 4.1. Voltage-Dependent Anion Channel (VDAC)

The three VDAC isoforms (1–3) control the flux of metabolites and calcium ions across the OMM, thus participating in the buffering of cytosolic Ca^2+^. In a physiological context, VDAC is considered a regulator of apoptosis. Upon apoptotic stimuli, VDAC1 undergoes oligomerization, interacts with pro-apoptotic proteins and promotes the release of cytochrome *c* to the cytosol, leading to apoptosis [106]. Enhanced Ca^2+^ flux through VDAC is generally associated with oxidative stress and the initiation of apoptosis. One would therefore assume that enhanced expression of VDAC is detrimental to tumor cells, and that VDAC isoforms are lost in most cancer cells. However, VDAC isoforms are often highly expressed in tumors, and this has been mechanistically linked to the role of VDAC in metabolic and apoptosis regulation [106,107,108]. It has been proposed that the switch from oxidative phosphorylation to glycolysis is consequent of VDAC1 binding to hexokinase (HK-II), a cytosolic enzyme, which catalyzes the phosphorylation of glucose to glucose-6-phosphate [109,110]. In various cancer types, cytosolic HK-I and HK-II levels are elevated with increased translocation to the OMM. HK binding to VDAC1, allows its direct access to mitochondrial ATP to induce glucose phosphorylation into glucose-6-phosphate (G-6-P), a rate-regulatory step in glycolysis. The coupling between VDAC1 and HK leads to elevated glycolysis, promoting cell growth [111,112]. In addition, studies have shown that VDAC1-HK prevents mitochondria-mediated apoptosis triggered by Bax or Bak [113] and that undocking of HK from mitochondria leads to cytochrome *c* release. Mitochondria-bound HK is also found to attenuate ROS induced apoptosis by reducing mROS generation [114]. In this context, HK bound to VDACs protects against oxidative stress by minimizing permeabilization of the OMM and subsequent release of cytochrome *c*, an important scavenger of O_2_^•−^ [115]. Consequently, in tumor cells with elevated levels of HK bound to VDAC1, apoptosis is suppressed, and proliferation is facilitated. Thus, the disruption of the HK-VDAC1 interaction should facilitate cell death and has been investigated as a potential therapeutic strategy [108]. Although seemingly counterintuitive, several other compounds that appear to block VDAC channel conductance also induce apoptosis, presumably due to their inhibition of substrate flux in and out of mitochondria, including ATP/ADP.

Since cancer cells generally evade VDAC dependent apoptosis and associated oxidative stress, fewer studies have focused on its role as a regulator of the [Ca^2+^]m-dependent changes in sub-lethal mROS involved in redox signaling. Because VDAC mediates a switch toward glycolysis and affects ATP flux from the mitochondria, ETC function and ETC-dependent mROS production are likely also affected by changes in VDAC expression and regulation by HK binding. Moreover, evidence suggests that VDAC1 acts as a preferential release channel for the hydrophilic O_2_^•−^ anion, produced during respiration into the IMS [116], and could hence act as regulator of redox signaling in cancer cells by promoting mROS efflux. In this review we focus on the IMM proteins involved in regulating [Ca^2+^]m in the mitochondrial matrix, which is the primary site of mROS production, but refer the reader to several review articles discussing the role of VDACs in cancer [12,106,108,112].

### 4.2. Mitochondrial Ca^2+^ Uniporter (MCU)

The mitochondrial Ca^2+^ uniporter (MCU), together with NCLX, regulate matrix [Ca^2+^]m. In most cancers levels of MCU are elevated, and while regulation of MCU expression has not been widely studied, the Cancer Genome Atlas (TCGA) demonstrates that the MCU gene is amplified in some tumor types [117]. A potential transcriptional regulatory mechanism includes the loss of microRNA down-regulation [117]. The MCU-ROS interplay was identified as an important regulator of triple negative breast cancer (TNBC) cell growth and metastasis. Tosatto *et al*. showed that in TNBC, MCU expression correlated with tumor size and lymph node infiltration [118]. Upon MCU knockdown, migration and invasion of breast cancer cells were decreased, and *in vivo* tumor growth abrogated. The authors showed that MCU knockdown correlated with a decrease in [Ca^2+^]m uptake, a decline in ROS production and HIF1-α expression [118]. It was demonstrated that MCU knock-down decreases NADH levels and ATP production, suggesting that MCU regulates the production of NADH reducing equivalents necessary for ATP production at the ETC, which is also the major site of ROS production in mitochondria [118]. In contrast, another study demonstrated that MCU or MICU1 knock-down had no effect on ROS production, although [Ca^2+^]m was reduced, in the TNBC cell line MDA-MB-392. However, the investigators noted that loss of MCU and MICU1 expression significantly affected cell survival of normal human mammary epithelial cells (HMECs) [119]. Effects on migration and invasion were not assessed in this study and one might conclude from the above that MCU is primarily required for migration, invasion and metastasis of TNBC cells, rather than cell survival or proliferation. This also correlates with observations that MCU expression is further elevated in metastatic disease in many breast cancer subtypes [120]. MCU expression is upregulated in hepatocellular carcinoma (HCC) cells from metastatic samples, with a significant correlation between high MCU expression and poor patient prognosis [121]. The investigators confirmed that Ca^2+^ uptake into mitochondria was enhanced and promoted ROS production as a consequence of enhanced MCU expression in MHCC97H cell lines. Here, MCU expression resulted in a downregulation of the NAD^+^/NADH ratio, which decreased SIRT3 activity, a NAD^+^-dependent mitochondrial deacetylase. In turn, this inhibited the activity of the mitochondrial superoxide dismutase SOD2, a SIRT3 substrate, which is essential for mitochondrial matrix superoxide scavenging. Buffering [Ca^2+^]m and subsequent reduction in mROS decreased the migration and invasion of HCC cells and metastasis formation in vivo. Further, mROS activates the Jun N-terminal kinase (P-JUNK) pathway to drive HCC migration [121]. In this study, the investigators also showed that highly metastatic cells have an increase in basal [Ca^2+^]m levels compared to normal hepatocytes [121].

Reciprocally, ROS can also directly affect MCU activity. A recent study from Dong et al., identified that the conserved cysteine 97 (Cys-97) is the only reactive thiol in human MCU that undergoes S-glutathionylation [122]. They demonstrated that in response to exposure of inflammatory signals such as lipopolysaccharide (LPS) or hypoxia-derived ROS, the MCU central component senses mitochondrial ROS via Cys-97 oxidation and exhibits a higher order oligomer formation. This [Ca^2+^]m –mROS positive feed-back loop is independent from the other regulatory proteins of the MCU complex and leads to higher mitochondrial Ca^2+^ uptake and mROS production [122]. 

### 4.3. Mitochondrial Ca^2+^ Uniporter (MCU) Regulators

ROS production in the mitochondria is affected not only by MCU expression but also by MCU regulators, including MCUR1, MICU1 and MICU2. 

Similar to the enhanced MCU expression in HCC discussed above [121], the same group showed that MCUR1 expression was also increased in HCC cells, and promotes proliferation and survival of HCC cells [124]. The MCUR1-mediated [Ca^2+^]m uptake was MCU dependent and resulted in elevated mROS production. This stimulated degradation of the tumor suppressor p53 via activation of the AKT/Mdm2 (murine double minute 2) pathway [124]. When Akt phosphorylates Mdm2 at Ser166 and Ser186, the ubiquitin-ligase activity of Mdm2 is enhanced and indirectly suppresses p53-mediated apoptosis and enhances the activity of cell cycle-related molecules [127]. MCUR1 was shown to not only promote survival of HCC cells but also facilitate epithelial to mesenchymal transition (EMT) and metastasis [128]. MCUR1 promoted in vitro migration and invasion and *in vivo* metastasis by inducing the expression of the EMT-transcriptional regulator Snail. MCUR1-dependent increase in [Ca^2+^]m and subsequent mROS generation activated the ROS/Nrf2/Notch1 pathway and elevated Snail expression. These phenotypes were reversed when [Ca^2+^]m was buffered with parvalbumin and when mROS was scavenged with mito-Tempo [128]. 

The absence of the gatekeeper protein MICU1 increases [Ca^2+^]m uptake by MCU and subsequently affects ROS production and apoptotic sensitivity [20]. A recent study showed that the phosphorylation of the N-terminal region of MICU1, by a pool of active mitochondria-localized Akt, affects its maturation and stability. Although the localization of kinases without canonical mitochondrial targeting sequences remains controversial, this phosphorylation of MICU1 was shown to alter the [Ca^2+^]m accumulation, leading to ROS production and tumor progression [129]. This highlights that pro-tumorigenic signaling pathways can alter the activity of mitochondrial Ca^2+^ transport to further manipulate mitochondrial redox signals. 

The above studies demonstrate that increases in [Ca^2+^]m by regulated activation of MCU regulators can lead to mROS production necessary for pro-tumorigenic signaling. However, if [Ca^2+^]m and subsequent mROS production exceeds a threshold that elicits deleterious effects in mitochondria, consequences of hyperactivation of mitochondrial Ca^2+^ uptake can lead to mitochondrial Ca^2+^ overload and cell death. There are several examples in the literature that demonstrate that tumor cells also adapt to prevent these deleterious surges in [Ca^2+^]m by regulating the MCU gatekeepers, MICU1, and MICU2. In ovarian cancer, the MICU1 protein is highly expressed and this is associated with a poor prognosis and chemoresistance [126]. MICU1 knockdown was shown to inhibit tumor growth and increased cisplatin efficacy. Mechanistically, MICU1 knockdown activated pyruvate dehydrogenase (PDH), and induced a switch from glycolysis to oxidative phosphorylation. MICU1 knockdown consequentially generated mROS to levels that induced oxidative stress in the mitochondria, leading to apoptosis. In pancreatic cancer cells, Chen *et al.* demonstrated that enhanced expression of the histidine triad nucleotide-binding (HINT2) protein elevates [Ca^2+^]m levels and subsequent ROS production leading to apoptosis [125]. HINT2 expression is commonly decreased in pancreatic cancer and is associated with poor prognosis. Upon HINT2 overexpression, Chen et al. observed lower levels of MICU1 and MICU2 and upregulation of EMRE levels [125]. HINT2 therefore sensitizes cells to apoptosis by altering the expression of MCU regulators, while cancer cells suppress this by downregulating HINT2 expression. In a similar way, overexpression of mitofusin-2 downregulates MICU1 and MICU2 expression in HCC [130]. Mitofusin-2 overexpression increases intracellular ROS and Ca^2+^ transfer from the ER to mitochondria, leading to cell apoptosis. 

The above studies demonstrate that MCU gatekeepers can be regulated transcriptionally and at the post-translational level to fine tune [Ca^2+^]m and subsequent mROS pro-tumorigenic signaling, while limiting pro-apoptotic mROS surges. It is clear that the role and regulation of MCU and its regulators is likely cancer-type specific. For example, Hall *et al.* failed to observe a change in ROS production or the survival of breast cancer cell lines under stress condition following MICU1 knockdown [119]. More work is therefore required to determine if therapeutic targeting of the MCU regulators is a valid approach for certain tumor types. 

### 4.4. Mitochondrial Na^+^/ Ca^2+^/Li^+^ Exchanger (NCLX (SLC8B1))

The NCLX-ROS interplay has not been examined in cancer. However, links between changes in NCLX expression, altered [Ca^2+^]m and mROS production have been developed in other pathologies [131]. By extruding [Ca^2+^]m from the mitochondrial matrix, NCLX is able to affect OXPHOS and mROS formation [29,132]. As expected, a loss in NCLX function results in reduced [Ca^2+^]m efflux and increased cytosolic Na^+^ levels, which results in TCA cycle activation, changes in NADH/NAD^+^ ratios and mROS production [132,133]. It was found that NCLX expression was induced in rat aortic endothelial cells in response to high glucose stress. The authors found that NCLX expression protected cells from mitochondrial oxidative stress in response to high glucose exposure, as knockdown of NCLX increased [Ca^2+^]m levels, leading to elevated ROS production and increased NLRP3 inflammasome activation [133]. Altering NCLX expression can also lead to redox signaling that inhibits Ca^2+^ entry to the cytoplasm via SOCE. Elevations in mitochondrial H_2_O_2_ in response to NCLX knockdown were shown to result in inhibitory oxidation of Cys-195 of ORAI1 to reduce SOCE [134]. Recently, the role of NCLX has been studied using tissue specific knockout mice. Tamoxifen-induced deletion of NCLX in adult mouse hearts caused sudden death, which was attributed to Ca^2+^ overload in mitochondria causing an increase in mitochondrial superoxide generation, and subsequent cell death [30]. The above studies show that altering mitochondrial Ca^2+^ extrusion by NCLX can influence ROS production in pathological conditions. Interestingly, while not directly linked to changes in NCLX expression in the tumor cells tested, the NCLX inhibitor CGP37157 was able to sensitize chemoresistant melanoma, osteosarcoma, and prostate cancer cells to pro-apoptotic tumor necrosis factor-related apoptosis-inducing ligand (TRAIL) [135,136]. Moreover, CGP37157 was shown to influence mitochondrial shape, by inducing mitochondrial Ca^2+^ overload, affecting Mfn1 degradation, mitochondrial fusion and apoptosis in prostate cancer cells [137,138]. It is unclear if this is directly due to NCLX inhibition, or off-target effects of the drug on other channels and transporters. Interestingly, it has been reported that CGP37157 prevents neurotoxicity in response to the chemotherapeutic salinomycin, while this protective effect was not observed when head and neck squamous cell carcinoma cells were co-treated with salinomycin and CGP37157 [139]. It is possible that NCLX expression changes between neurons and cancer cells differ to affect the activity of CGP37157 on cell viability. However, further work is required to elucidate if altered NCLX expression contributes to the [Ca^2+^]m-mROS axis in cancer and whether this also represents a viable pharmacological target.

## 5. Perspectives and Conclusion

Mitochondria are intracellular organelles that play key roles in energy production and in the regulation of many cellular processes from cell signaling to death. The ETC is the major source of ATP in the cell and its activity is coupled with the generation of mROS that are finely maintained at physiological levels by highly efficient mitochondrial antioxidant systems. In the past few years, several studies have demonstrated that mROS are essential for physiological signaling, but when the balance between mROS production and elimination is altered, by either overproduction of mROS or impairment of the antioxidant defensive mechanisms, mitochondrial dysfunction occurs. One important mechanism regulating mitochondrial mROS homeostasis in cancer cells is through alterations in [Ca^2+^]m by mitochondrial Ca^2+^ transport. While we only touch on a few examples from an ever-expanding literature, we highlighted several examples where Ca^2+^ within the mitochondria has an important regulatory function on mROS production and redox signaling events that are beneficial to tumor cell survival and progression. As mentioned in Section 3, [Ca^2+^]m influences the activity of several TCA enzymes in the mitochondria, which likely drives mROS production. As described above, several studies have demonstrated that manipulation of [Ca^2+^]m results in changes in the NAD^+^/NADH ratios. However, direct evidence that [Ca^2+^]m alters TCA cycle enzyme or ATP synthase activity in cancer cells requires further attention. This is of particular importance in the context of genetic aberrations that may influence the expression and activity of PDH and TCA cycle enzymes, and in the context of other metabolic changes, such as substrate flux, that could influence the action of [Ca^2+^]m on mitochondrial metabolism. The discovery of MCU and its regulatory proteins opens a new era in the study of MCU-mediated mitochondrial Ca^2+^ homeostasis in cancer. Currently, the available candidate agents targeting MCU or its regulators in cancer chemotherapy are still burgeoning [141,142], and much progress needs to be made in order to test their efficacy as anti-cancer agents to improve clinical chemotherapeutic outcomes in cancer patients. As illustrated in Table 2, the use of these agents in cancer studies has been limited, and the information garnered from this work is still in its infancy. Moreover, the consequences of these inhibitors on metabolism and mROS generation and signaling regulated by [Ca^2+^]m have not been explored, opening exciting avenues of investigation.

## Figures and Tables

**Table 1:** Example studies demonstrating the role of [Ca^2+^]m regulators located in the inner mitochondrial membrane (IMM) on mROS production and redox signaling in cancer.

Cancer Type	[mCa^2+^] Regulator (Expression in Tumor Specimens)	Cell line (Normal/Control Cells)	[Ca^2+^]_m_ Phenotype	mROS Phenotype	Cellular Phenotype	Reference
**Breast Cancer**	MCU (elevated in TNBC)	TNBC cell lines: MDA-MB-231 MDA-MB-468 BT-549 (none)	MCU knock-down results in decreased mCa^2+^ uptake induced by ATP; MCU expression increases [Ca^2+^]_m_ transients and cytosolic Ca^2+^ buffering by mitochondria.	MCU knock-down results in decreased mito O_2_^∙−^ mitochondrial GSSG/GSH, and H_2_O_2_, leading to decreased HIF-1α activation.	MCU knock-down results in decreased migration, invasion, clonogenic potential, in vivo tumor growth and metastasis; no effect on proliferation, cell cycle or cell death.	[118]
	MCU (elevated in ER negative and basal-like breast cancers)	MDA-MB-231	Effects on [Ca^2+^]_m_ not tested. MCU knock-down had no major effect on cytosolic Ca^2+^.	Not investigated	MCU knock-down results in potentiation of cell death by ionomycin; no effect on proliferation.	[123]
	MCU, MICU1 (high MCU, low MICU1 associated with poor survival of breast cancer patients)	MDA-MB-231	MCU knock-down results in decreased mCa^2+^ uptake induced by ATP; MCU dominant negative (DN) expression decreases the integrated area of response induced by ATP.	No effect on MitoSox following MCU or MICU1 knock-down in MDA-MB-231	MCU knock-down increases AMPK activation. MCU or MICU1 knock-down, or MCU-DN had no effect on clonogenic survival in response to therapy-related stress in MDA-MB-231.	[119]
		(HMEC)	MICU1 knock-down increases peak amplitude of [Ca^2+^]_m_ uptake; increases integrated area of response induced by ATP.		MCU knock-down in HeLa affects cell viability in response to ceramide. MCU and MICU1 knock-down in HMEC affects cell viability in response to ceramide.	
	MCU (elevated expression correlates with metastatic disease)	MDA-MB-231	MCU inhibition by RuR or MCU knock-down decreases [Ca^2+^]_m_ induced by serum; decreases SOCE induced by thapsigargin.	Not tested	MCU inhibition by RuR or MCU knock-down decreases migration.	[120]
**Cervical Cancer**	MICU1	HeLa (human endothelial cells [HEC])	MICU1 knockdown in HeLa and HEC results in increased [Ca^2+^]_m_ under resting conditions; no effect on the peak [Ca^2+^]_m_ or [Ca^2+^]_cyto_ induced by histamine.	MICU1 knockdown in HeLa and HEC results in increased basal mROS.	MICU1 knockdown in HeLa increases ceramide induced cell death; no effect on proliferation.	[20]
					MICU1 knockdown in HEC increases LPS and cycloheximide induced cell death; decreases migration	
	MCU	HeLa	Not tested	Not tested	MCU knock-down in HeLa affects cell viability in response to ceramide	[119]
**Liver Cancer**	MCU, MICU1 (high MCU, low MICU1 expression in HCC compared to matched normal)	MHCC97H, SMMC7721, BEL7402 (Normal hepatocyte HL-7702)	HCC have increased basal [Ca^2+^]_m_ compared to normal hepatocyte.	MCU knock-down in HCC decreased mROS and total ROS.	MCU knock-down in HCC decreased migration, invasion and *in vivo* metastasis.	[121]
			MCU knock-down in HCC decreased [Ca^2+^]_m_ in response to histamine.			
			MCU expression in HCC increased [Ca^2+^]_m_ in response to histamine.	MCU expression in HCC increases mROS and total ROS, leading to ROS-dependent JNK activation.	MCU over expression in HCC increased migration, invasion and *in vivo* metastasis.	
			MICU1 knock-down in HCC increased [Ca^2+^]_m_ in response to histamine.			
	MCUR1 (increased expression in HCC compared to matched normal)	BEL7402, MHCC97H (none)	MCUR1 knock-down decreased [Ca^2+^]_m_ in response to histamine.	MCUR1 knock-down decreased mROS and total ROS.	MCUR1 knock-down increased apoptosis; decreased proliferation, clonogenic potential, and *in vivo* tumor growth.	[124]
			MCUR1 overexpression in HCC increased [Ca^2+^]_m_ in response to histamine; which is abrogated by MCU inhibition with Ru360.	MCUR1 expression in HCC increases mROS and total ROS, leading to p53 inactivation via Akt/MDM2 pathway.	MCUR1 over-expression decreased apoptosis; increased proliferation, clonogenic potential, and *in vivo* tumor growth.	
**Pancreatic Cancer**	HINT2: regulator of MICU1/2, EMRE (HINT2 downregulated in Panc, decrease associated with poor prognosis)	BxPC-3, L3.6pl	HINT2 overexpression decreases MICU1 and MICU2 expression; increases EMRE.	HINT2 overexpression increased mROS.	HINT2 overexpression increased apoptosis, decreased migration, invasion, clonogenic potential and tumor growth *in vivo*.	[125]
			HINT2 overexpression increased [Ca^2+^]_m_.			
**Ovarian Cancer**	MICU1 (high MICU1 expression associated with decreased patient survival; increased expression in chemoresistant OVCA specimens)	CP20, OV90 (normal fallopian tube and surface eptilelium cell lines: FTE188, OSE)	MICU1 knock-down increased [Ca^2+^]_m_ in response to cisplatin.	MICU1 knock-down increased mROS.	MICU1 knock-down decreases glycolysis in OVCA cells.	[126]
			Increased MICU1 expression correlates with pPDH. MICU1 knock-down restores PDH activity.		MICU1 overexpression enhances glycolysis in FTE188 and OSE.	

**Table 2:** Pharmacological targeting of [Ca^2+^]_m_ regulators of the IMM.

Target	Compound	Cellular Effects (Cancer Cells Tested)	Reference
MCU Inhibitor	Ruthenium Red/Ru360	Targets DXXE motif of MCU. Lacks specificity. Enhanced cytotoxicity in leukemia, HCC, breast and pancreatic cancer cells.	[120,124,140,141]
	Mitoxantrone (and analogs)	Targets DXXE motif of MCU. Not specific to MCU, has DNA intercalating activity, inhibits DNA topoisomerase II and cell proliferation. Used to treat prostate cancer, metastatic breast cancer, some leukemias	[141,142,143]
	KB-R7943	Inhibits Na^+^/Ca^2+^ exchanger (NCX1). Has anti-tumor activity, but not tested in context of MCU expression.	[144]
	DS16570511	MCU inhibitor—unclear mechanism. Not tested in cancer cells.	[145]
MCU Activator	Kaempferol	Lacks specificity/Mechanisms of MCU activation unclear. Pro-apoptotic. Anticancer properties observed in various tumor types.	[146,147,148]
NCLX Inhibitor	CGP37157 (benzothiazepine)	Off-target effects on other Ca^2+^ channels observed. Sensitizes tumor melanoma, osteosarcoma, and prostate cancer cells to pro-apoptotic stimuli.	[135,136,149]
	tetraphenylphosphonium	Demonstrated to inhibit mitochondrial Na^+^/Ca^2+^ exchange. Lacks demonstrated specificity toward NCLX.	[150]
	Cyclosporin A	NCLX inhibition at higher concentrations (IC_50_ = 2μM) than those required to inhibit mPTP.	[151]
	Verapamil	Ca^2+^ channel blocker, inhibits mitochondrial Na^+^ Ca^2+^ exchange, not specific toward NCLX.	[152]
	Amiloride analogs	Na^+^ channel blocker, not specific toward NCLX.	[153]
